# Long-Term Cognitive Outcomes of Esketamine Nasal Spray in Treatment-Resistant Depression: A Preliminary Report

**DOI:** 10.3390/ph18020173

**Published:** 2025-01-27

**Authors:** Matildes de Freitas Menezes Sobreiro, Paulo Sergio Panse Silveira, Vitor Breseghello Cavenaghi, Leandro Paulino da Costa, Bruno Pinatti Ferreira de Souza, Rachel Emy Straus Takahashi, Renato Vianna Marotta Starek, José Oliveira Siqueira, Renerio Fraguas

**Affiliations:** 1 Grupo de Interconsultas, Departamento e Instituto de Psiquiatria, Hospital das Clínicas Faculdade de Medicina da Universidade de São Paulo, São Paulo CEP 05403-903, SP, Brazil; cavenaghi.vb@gmail.com (V.B.C.); pc.leandro@uol.com.br (L.P.d.C.); brunopinatti@gmail.com (B.P.F.d.S.); chel_est89@hotmail.com (R.E.S.T.); 2 Departamento de Patologia, Faculdade de Medicina da Universidade de São Paulo, São Paulo CEP 05403-903, SP, Brazil; paulo.silveira@fm.usp.br (P.S.P.S.); siqueira@usp.br (J.O.S.); 3 Centro de Desenvolvimento de Ensino Médico—CEDEM, Faculdade de Medicina, Universidade de São Paulo, São Paulo CEP 05403-903, SP, Brazil; renatostarek@gmail.com; 4 Laboratório de Investigação Médica-21 LIM-21, Grupo de Interconsultas, Departamento e Instituto de Psiquiatria, Hospital das Clínicas Faculdade de Medicina, Universidade de São Paulo, São Paulo CEP 05403-903, SP, Brazil; 5 Divisão de Psiquiatria e Psicologia do Hospital Universitário, Universidade de São Paulo, São Paulo CEP 05403-903, SP, Brazil

**Keywords:** ketamine, esketamine, neuropsychological, cognitive, long term, treatment-resistant depression, depression

## Abstract

**Background/Objectives**: Ketamine/esketamine has a rapid/robust antidepressant effect on treatment-resistant depression (TRD). However, its long-term cognitive effects remain unclear. In this study, we investigated the potential cognitive effects of an esketamine spray on a series of TRD patients. **Methods**: We evaluated the cognitive performance of eight TRD patients subjected to an esketamine nasal spray as an adjunct treatment for six months. Cognitive assessments were performed before treatment initiation (T0) and at three (T3) and six (T6) months by an experienced neuropsychologist using a comprehensive neuropsychological battery. Depression severity was assessed by the Montgomery–Åsberg Depression Rating Scale. Changes in cognitive performance were analyzed by determining the bias between time points. To investigate the association between the severity of depression and performance on cognitive tests, we used correlation with correction for repeated measures and regression analysis with a general linear mixed model. We used the Tukey method to compare three estimates and the Dunnett method to compare two estimates. **Results**: Improvements in at least one test from T0 to T6 were found for attention, memory, and the executive functions of working memory, set-shifting, and inhibitory control. Most of the improvements had occurred by T3, but working memory and set-shifting improvements were significant only at T6. The severity of depression decreased significantly from T0 to T6, and most cognitive improvements were correlated with an improvement in depression severity. No test indicated a worsening of cognitive performance from T0 to T6. **Conclusions**: Our results suggest that the cognitive performance of TRD patients improved with long-term adjunct treatment with an esketamine nasal spray. Confirmatory studies are necessary.

## 1. Introduction

Depression, especially treatment-resistant depression (TRD), may impair cognitive functions [1]. Attention, executive functions, and verbal memory exhibit consistent impairment, even in remitted major depressive disorder (MDD) patients [2]. Patients with TRD have been shown to perform worse than those with first-episode depression on tests assessing executive function, including the Trail Making Test B, the modified Wisconsin card sorting test (M-WCST), and the Tower of Hanoi test [3]. Moreover, in addition to impairments in verbal working memory, mildly reduced performance has been reported across all neurocognitive domains in TRD patients [4]. It has been proposed that the cognitive impairment may result from the progression of depression [3]; may be secondary to medications used to treat depression, such as benzodiazepines [5]; and that cognitive impairment is a risk factor for depression non-responsiveness [6]. Pharmacological treatment of cognitive impairment in TRD patients remains a challenge. Most antidepressants, including tricyclic antidepressants, selective serotonin reuptake inhibitors, and serotonin–norepinephrine reuptake inhibitors, have not been shown to exhibit procognitive effects [7]. Bupropion [8] and duloxetine [9] have shown positive effects on cognition, but the existence of a specific procognitive effect has to be proven. Actually, a meta-analysis found that vortioxetine was the only antidepressant that produced an improvement in the Digit Symbol Substitution Test in patients with depression (not specifically TRD) [10]. Besides traditional antidepressants, pharmacological interventions to improve cognitive function in patients with depression are only experimental. In that context, an initial study has reported procognitive effects of erythropoietin on verbal recall and recognition in patients with TRD [11].

Both the racemic presentation of ketamine [12] and its S-enantiomer, esketamine, have demonstrated robust efficacy in treating TRD [13]. Considering the cognitive effects of ketamine/esketamine as an antidepressant, systematic literature reviews have reported favorable results [14], without findings of negative effects of IV ketamine on cognition [15]. After a single infusion, the use of ketamine for TRD has been associated with no adverse neurocognitive effects [16] or even a slight improvement in sustained attention and response control [17]. A study involving six intravenous infusions of ketamine (0.5 mg/kg) over a 12-day period in 84 patients found a significant improvement from baseline in the speed of processing and verbal learning scores at day 13 and speed of processing at day 26, but not in other cognitive performance measures [18]. Long-term therapeutic use, specifically 44 weeks of esketamine treatment, has also shown favorable results regarding visual and verbal learning, memory, working memory, and executive function [19]. However, impairments have been reported in processing speed 24 h after a single infusion and in verbal memory 24 h after six infusions of ketamine [20]. Additionally, a slowing of performance on simple and choice reaction time tests of the CogState has been reported after the long-term treatment of patients aged ≥ 65 years [19]. Additionally, cognitive deficits have been described in frequent users of ketamine for “recreational purposes”, including decreased performance in spatial working memory and pattern recognition memory tasks [21]. A cognitive impairment has also been reported shortly after the administration of a sub-anesthetic dose of ketamine in healthy volunteers [22,23]. In this context, we conducted this study to investigate specifically the cognitive performance outcomes of patients with TRD after six months of adjunct treatment with an esketamine nasal spray. Additionally, we investigated the correlations between improvements in depression and changes in cognitive performance.

## 2. Results

We present data from eight TRD patients with at least two neuropsychological assessments during six months of adjunct intranasal esketamine treatment. Initially, the 12 patients from the “Expanded Assess Program” of our site agreed to participate in the study and were subjected to the baseline neuropsychological assessment. However, four of them were not subjected to additional neuropsychological evaluations. Two of them said they did not want to make additional neuropsychological assessments; one opted to continue the treatment with ketamine in a center closer to his home, and one withdrew from the study because he was led to the emergency room by a friend before the second neuropsychological assessment. Possibly because of the improvement in his psychomotor skills, the patient told a friend about his suicidal thoughts. Although reporting that there was not a worsening in the suicidal ideation in our assessment, the friend took him to the emergency room, and that was a criterion to withdraw from the study. The demographic characteristics of patients are shown in Table 1.

In Table 2, we present the descriptive results for the severity of depression and neuropsychological assessments at baseline (T0), three months (T3), and six months (T6) after beginning esketamine treatment. Patients exhibited significant improvements in their performance from T0 to T6 across 7 out of 21 tests, namely, TMT-A; RAVLT—correct and RAVLT—immediate; Logical Memory II; Digit Span Backward; Stroop Color; and WCST—total correct (Table 3, Table 4 and Table 5). No test showed worsening from T0 to T6; the remaining 14 tests did not significantly change during this period.

Significant improvements from T0 to T3 were observed in 9 of the 21 tests, including TMT-A; Digit Symbol-Coding; RAVLT—correct and RAVLT—30 min delay; Logical Memory I and II; Stroop Color; WCST perseverative errors; and WAIS—vocabulary (Table 3, Table 4 and Table 5). However, the improvement in performance from T0 to T3 on 4 tests was not maintained when assessed over the interval from T0 to T6, including the RAVLT—30 min delay, Logical Memory I, WCST perseverative errors, and WAIS—vocabulary. Intriguingly, after improving from T0 to T3, performance on Symbol Coding that was impaired from T3 to T6 was not different from the level it was at T0 (Table 3, Table 4 and Table 5). On the other hand, two tests, Digit Span Backward and WCST—total correct, showed significant improvements from T0 to T6 but not from T0 to T3. Specifically, in the Digit Span Backward test, the significant improvement occurred between T3 and T6, whereas in the WCST, the improvement was more gradual from T0 to T6. All analyses of this study were corrected by the Bonferroni test and adjusted for gender, age, and educational level.

The association between depression (total MADRS score) and each neuropsychological test was assessed using repeated measures correlation and linear regression, with each patient serving as their own control (applying the random effect model). While these are two distinct models—one involving a correlation (which is not directional) and the other modeling the effect of depression on the test results—their conclusions mostly align (Table 6, Table 7, Table 8 and Table 9). The respective correlation coefficients and regression slopes with 95% confidence intervals and statistical significance are shown in Table 6, Table 7, Table 8 and Table 9. The analysis revealed significant correlations between a decrease in depression severity and improvement in performance from T0 to T3 on the following tests: TMT-A, Digit Symbol-Coding, Logical Memory II, and WCST perseverative errors (Table 6, Table 7, Table 8 and Table 9). However, the improvements from T0 to T3 on RAVLT—correct, RAVLT—30 min delay, Logical Memory I, Stroop Color, and WAIS—vocabulary were found to be independent of changes in depression severity. The improvements in performance from T0 to T6 exhibited a correlation with a reduction in the severity of depression for the following tests: TMT-A, Logical Memory II, Digit Span Backward, and Stroop Color. However, the improvements observed from T0 to T6 on RAVLT—correct and RAVLT—immediate, as well as on WCST—total correct, did not demonstrate any significant correlation with changes in the severity of depression (Table 6, Table 7, Table 8 and Table 9). Considering the two tests, Digit Span Backward and WCST—total correct, that showed significant improvements from T0 to T6 but not from T0 to T3; the changes in the Digit Span Backward test, between T3 and T6, were correlated with changes in depression severity, whereas changes in the WCST—total correct between T0 and T6 were not correlated with changes in depression severity (Table 6, Table 7, Table 8 and Table 9).

## 3. Discussion

In this study involving eight individuals diagnosed with TRD, we investigated the impact of a six-month adjunct treatment with an esketamine nasal spray on cognitive performance. Employing a comprehensive neuropsychological battery administered by an experienced neuropsychologist, our findings revealed significant improvement from T0 to T6 in at least one test within five of the eight assessed cognitive domains. Most of the improvements detected at T6 had already occurred by T3. However, we found that an improvement in some cognitive abilities may require more than three months, as indicated by two tests. Conversely, we also found that the early cognitive improvements already achieved at T3 may not be sustained later at T6. Importantly, no impairments at T6 were detected in any tests compared to T0. Our results also indicated that most changes in cognitive performance were related to changes in depression severity. Considering the inherent limitations of the design and sample size of our study, these results should be regarded as hypothesis-driven. Notwithstanding, we discuss these findings in light of the existing literature data.

According to at least one test, the five cognitive domains that improved from T0 to T6 were attention, memory, and the executive functions of working memory, set-shifting, and inhibitory control.

Attention improvement, as assessed by the TMT-A, demonstrated significance at T3, and a further improvement occurred from T3 to T6. Our results agree with short-term studies (*n* = four manuscripts) [17,24,25,26] that reported an improvement in attention with ketamine treatment in TRD patients, whereas one manuscript reported no changes [20]. We did not find studies reporting a worsening of attention with ketamine treatment as an antidepressant. Chen et al. (2018) found an improvement in attention, as assessed by higher correct responses and lower omissions in the Go/No-Go task, on day three and day 14 after ketamine administration in a study administering a 0.5 mg/kg ketamine (*n* = 24) or 0.2 mg/kg ketamine infusion (*n* = 23) [17]. An improvement of attention, also assessed by the TMT-A, but as soon as 14 days after baseline, was reported by Basso et al. (2020) in 25 patients referred for electroconvulsive therapy (ECT) who received a total of six to nine sub-anesthetic doses (0.5 mg/Kg) of intravenous infusions of ketamine thrice a week [25]. Phillips et al. (2022) also found improvements in attention and processing speed assessed by the TMT-A and the Stroop congruent condition in 38 TRD patients after seven infusions of ketamine and one double-blind infusion followed by two weeks of thrice-weekly open-label repeated ketamine infusions [26]. Petropulu et al. (2023) found an improvement in complex attention using the NIHToolbox Cognition Battery in 66 TRD patients treated with four ketamine infusions, 0.5 mg/kg, administered 2–3 times a week [24]. Differently, Shiroma et al. (2014), using the CogState battery, reported no changes in attention after six IV infusions of ketamine over three weeks involving 15 TRD patients [20]. It should be noted that the improvement in attention in our patients was significant in only one of the four tests used to assess it. Nevertheless, the improvement was significant from T0 to T3 and significant from T3 to T6. It is also noteworthy that it may start as early as after 14 days of ketamine treatment, as found by Basso et al. [25]. Altogether, adjunct treatment with esketamine for TRD seems to impact attention positively. However, given the limited number of studies, it is not possible to exclude that future studies may uncover a possible negative impact of ketamine on attention in some patients.

The improvement in memory from T0 to T6 was detected by the RAVLT correct answers, by the RAVLT immediate, and by the Logical Memory II test. Immediate memory refers to the immediate retention and, differently from working memory, it functions as a passive storage system with minimum processing and manipulation of information. Improvements in the RAVLT correct answers and in the Logical Memory II test occurred significantly from T0 to T3 and from T3 to T6. The improvements in immediate memory and delayed episodic memory found in our patients are in line with short-term studies (*n*= nine manuscripts) employing ketamine as an antidepressant [16,18,20,24,25,26,27,28,29]. Three of these manuscripts are from the same group and the samples overlapped [18,28,29]. One short-term study found no changes in its assessments of memory [30], and at least three manuscripts of those reporting memory improvement also reported no changes in any other test assessing memory [18,28,29]. In contrast, an impairment in memory was found in two short-term studies [25,31]. Among the studies with positive findings, Shiroma et al., in their study of 2014 (mentioned above), reported an improvement in visual memory assessed with the CogState battery [20]. Improvements in verbal and visual learning were reported by Murrough et al. in their second study on this topic (2015) using the MATRICS Consensus Cognitive Battery (MCCB) in 43 TRD patients seven days after a single ketamine infusion [16]. An improvement in verbal learning has been reported by Zhou et al. (2018) and Zheng et al. (2019) after a series of six intravenous IV infusions of ketamine over a 12-day period, as assessed by the MATRICS Consensus Cognitive Battery at the baseline and days 13 and 26, either in the sample including bipolar and unipolar patients (*n* = 74) [18,28] or in the subsample of unipolar patients (*n* = 64) [28]. After six intravenous ketamine infusions in TRD patients, Liu et al. (2019) found improvements in verbal learning, as assessed with the MATRICS Consensus Cognitive Battery, on day 13 but not on day 26 in 30 patients with anxious depression but not in 20 non-anxious people [29]. Basso et al. (2020) (mentioned above) found improvements in visual memory assessed by the RAVLT after six to nine ketamine infusions [25]. Araújo-de-Freitas et al. (2021), using the RAVLT, the WAIS-III Digit Span Forward, and the Corsi Block-tapping Task Forward tests, found improvements in short-term memory, immediate recall, immediate recovery, and delayed recall in an open trial, 24 h and one week after a single infusion of ketamine or esketamine in 51 TRD patients [27]. Phillips et al. (2022) (mentioned above) found an improvement in verbal memory, as assessed by the California Verbal Learning Test-short form, after seven infusions of ketamine [26]. Zavaliangos-Petropulu et al. (2023) (mentioned above) found an improvement in episodic memory after the first and fourth ketamine infusions [24]. Our study also aligns with the only long-term study conducted to date [19]. In that study, SUSTAIN-2, a phase 3, open-label, multicenter study, Wajs et al. (2020) found an improvement in verbal learning in week 44 of the maintenance phase in 197 TRD patients [19]. SUSTAIN-2 administered esketamine intranasally twice a week in the 4-week induction phase, then weekly or every other week to responders in the 48-week optimization/maintenance phase [19]. It should be mentioned that studies reporting a memory improvement also found no improvement in memory, depending on the instrument or type of memory. No improvements in visual learning and memory were reported by Zhou et al. (2018) (mentioned above), Liu et al. (2019) (mentioned above) and Zheng et al. (2019) (mentioned above) [18,28,29]. Also, no significant changes have been reported in autobiographical and episodic memory in 28 patients with uni- or bipolar TRD after three weeks with either three or six ketamine infusions [30]. Notwithstanding the aforementioned relatively favorable profile of ketamine’s effect on memory after short- and long-term use as an antidepressant, some concerning data based on its administration in healthy volunteers, its use by depressed patients, and its impact on abusers deserve careful consideration. Impairments in delayed recall and in the learning of spatial and verbal information have been reported shortly after ketamine administration in healthy volunteers [23]. Considering depressed patients, an impairment in memory recall was found by Murrough et al. in their first study on this topic (2013), 40 min. after a single administration of IV ketamine in 25 TRD patients, as assessed with the Hopkins Verbal Learning Test (HVLT)–Delayed Recall, while no impairments in HVLT learning and category fluency were found [31]. The authors found that decreased cognitive performance was associated with a non-response to the antidepressant effects of ketamine [31]. An impairment in verbal memory was also found in the study by Basso et al. (2020) in delayed recall 14 days after baseline (small effect) in hospitalized patients with indication of ECT [25]. The authors considered this possible negative impact preferable to the effect of ECT on cognition [25]. The potential negative impact of ketamine on memory has also been described in ketamine abusers. Impairments in both source memory and item recognition have been found in ketamine abusers compared to poly-drug controls [32]. Based on animal data, it has been proposed that learning and memory deficits after long-term ketamine administration may result from a reduced expression and decreased phosphorylation of hippocampal postsynaptic membrane GluA1- containing AMPA receptors [33]. However, if confirmed, the potential negative impact of ketamine on memory, as reported by Basso et al. (2020), appears to be restricted to specific patient populations and conditions [25].

We found improvements in three executive functions: working memory, as assessed by the Backward Digit Span test; set-shifting, as assessed by the WCST; and inhibitory control, as assessed by the Stroop Color test. Some studies have analyzed specific executive functions, while others, including SUSTAIN-2, the long-term study by Wajs et al. (mentioned above) [19], and the study of Basso et al. [25], have reported improvements in executive function without analyzing specific functions. Phillips et al. (2022) found improvements in composite executive function by combining changes in the TMT-B, Stroop incongruent condition, and Digit Span Backward tests [26].

While it involves aspects of memory, the primary function of working memory is to manage and manipulate information rather than simply storing it; thus, it is considered an executive function. Five manuscripts describing short-term studies reported no changes in working memory [16,17,27,29,31], while one found no changes in spatial working memory but also reported an improvement in simple and complex working memory [20]. We did not find studies reporting working memory impairments following ketamine antidepressant treatment. Improvements in both simple and complex working memory, but not in spatial working memory, were found in the study by Shiroma et al. (2014) [20]. Improvements in working memory were found by Zavaliangos-Petropulu et al. (2023) using the NIHToolbox Cognition Battery [24]. Among the studies reporting no changes in working memory, Murrough et al. found no changes in the spatial span and the letter number task of the Wechsler Memory Scale in both of their studies (2013 and 2015; mentioned above) [16,31]; Chen et al. (2018) (mentioned above) also found no changes in working memory using a computerized working memory task [17]; Liu et al. (2019) found no changes in working memory assessed by the MATRICS Consensus Cognitive [29]; and Araujo de Freitas et al. (2021) found no changes using WAIS III Digit Span Backward and the Corsi Block-tapping Task Backward [27]. The only long-term study, SUSTAIN-2, also reported an improvement in the working memory of 197 patients on esketamine in week 44 of the maintenance phase [19]. Of note, the significant improvement in our patients occurred between the last three months of treatment and not in the first ones, suggesting that for some patients with depression, the working memory impairment may take a more extended period to be ameliorated.

The improvement in set-shifting in our TRD patients, as assessed by the WCST, is in line with one previous short-term study. Shiroma et al. (2020), in their later study, but not in their study of 2014 [20], found an improvement in attentional set-shifting assessed with CogState. They conducted a randomized trial in TRD patients who received six IV ketamine infusions for 12 days (*n* = 18) compared to IV midazolam, followed by a single IV ketamine infusion (*n* = 25) [34]. This possible effect of ketamine has been supported by animal studies showing its impact on the hippocampus–medial prefrontal cortex circuitry, correcting the cognitive set-shifting deficit caused by chronic unpredictable stress (Jett et al., 2015) [35].

The improvement in inhibitory control in our patients, as assessed by the Stroop Color test, improved from T0 to T3 and from T3 to T6. These results agree with two previous short-term studies [17,24], while no change was reported by Araujo de Freitas et al. (2021) [27]. An improvement in the Go/NoGo test was reported by Chen et al. (2018) on day 14 after a single ketamine infusion in 71 TRD patients [17]. Zavaliangos-Petropulu et al. (2023) found an improvement in the Flanker Inhibitory Control and Attention test using the NIHToolbox Cognition Battery [24]. Studies have described neurofunctional mechanisms probably related to the effect of ketamine on inhibitory control. Sahib et al. (2020) found that the performance on the Go/NoGo test of TRD patients at baseline did not differ from controls nor improved with four IV ketamine infusions twice or thrice a week [36]. However, they found that ketamine infusions lead to a reduction in fMRI activity within the dorsolateral prefrontal cortex and the superior and inferior parietal lobules—brain regions associated with response inhibition [36]. Additionally, ketamine increased functional activation in the supplementary motor area during a response-inhibitory task in remitters compared to non-remitters and normalized it toward levels of activation similar to those of control subjects [36].

Aside from the improvements mentioned above, we found no improvement in performance at T6 compared to T0 in tests assessing intellectual ability, processing speed, and verbal fluency. No consistent changes in these domains have been reported in the literature. Murrough et al. (2013), reported no changes in category fluency [31], while Diamond et al. (2014) found an improvement in semantic fluency [30], and no changes in reasoning/problem-solving were reported in the later study by Murrough et al. (2015) [16].

The processing speed of our patients, as assessed with the Symbol Coding test, was the sole worsening performance found in our patients and occurred only during the last three months. Intriguingly, this significant worsening from T3 to T6 was preceded by an initial significant improvement from T0 to T3 such that the performance at T6 was no different from the baseline level. Short-term studies have shown either an improvement (*n* = 2) [16,29] or no changes (*n* = 3) [20,29,31] in the speed of processing with ketamine used as an antidepressant. Murrough et al. (2013), using a single intravenous infusion of ketamine and the category fluency, TMT-A, and BACS Digit Symbol tests as indicators of processing speed, found no changes 40 min after a ketamine infusion in 25 TRD patients [31]. Shiroma et al. (2014) also reported no changes in the speed of processing assessed with the CogState battery after six intravenous infusions over 12 days in 15 patients with TRD [20]. However, Murrough et al. (2015) found an improvement in processing speed in their later study on day seven after a ketamine infusion in 43 TRD patients [16]. Patients with anxious TRD, but not those with non-anxious TRD, exhibited a significant improvement in the speed of processing, as assessed with the MATRICS Consensus Cognitive Battery on days 13 and 26 after a series of six intravenous infusions of ketamine [29]. Considering long-term studies, our findings of a possible late decrease in the speed of processing align with the results of the SUSTAIN-2 study [19]. In the SUSTAIN-2 study, a slowing reaction time, as assessed by the Detection–Attention (simple) and Identification–Attention (choice) reaction time of the CogState Computerized Test Battery, was found after week 20, particularly among patients over 65 years of age [19]. Similar to our results, the impairment in cognitive performance in the SUSTAIN-2 study was restricted to the processing speed and presented after long-term treatment. The possibility of cognitive impairments secondary to ketamine antidepressant treatment for some groups of patients should be maintained by researchers considering the heterogeneity of cognitive impairment caused by TRD and interactions with other pharmacological agents used to treat depression [37].

Considering the time required to achieve a significant improvement, most of the cognitive improvements observed at T6 had already occurred at the T3 assessment. However, significant improvements in working memory (Digit Span Backward) and set-shifting (WCST, total correct) were only achieved at T6. Intriguingly, improvements from T0 to T3 in four tests, two assessing memory (RAVLT—30 min delay, Logical Memory I), one assessing set-shifting (WCST, perseverative errors), and one evaluating intellectual ability (WAIS—vocabulary) did not maintain significance when performance at T6 was compared to T0. This possible transient nature of the cognitive improvement could not be assessed in previous studies due to their short-term design.

A significant reduction in the severity of depression was observed among our patients over the six months of adjunct esketamine treatment. Notably, improvements from T0 to T6 in attention, delayed episodic memory, working memory, cognitive flexibility, and inhibitory control were correlated with a decrease in the severity of depression. However, improvements in the same period in abstract thinking, set-shifting, and immediate memory were not correlated with an improvement in depression severity. The correlation between cognitive improvement and changes in the severity of depression has not been consistent across studies. Various studies have reported a relationship between the amelioration of depression and cognitive improvement. Improvements in visual memory, simple working memory, and complex working memory, as assessed with the CogState battery after six IV infusions in 12 days in 15 patients with TRD, accounted for changes in the severity of depression [20]. Improvements in the speed of processing and verbal learning were both significantly mediated by changes in depressive symptoms from baseline to one day following the last infusion and from baseline to two weeks post-infusion in patients with unipolar [28] and bipolar depression [18]. In the study by Phillips et al. (2022), improvements in attention, processing speed, working memory, and visuospatial memory were dependent on improvements in depression [26]. However, an improvement of cognitive functions independent of a decrease in depression severity has been reported in various studies. An absence of a correlation has been reported between changes in neurocognitive function, including the speed of processing, verbal learning, and memory assessed with the MATRICS Consensus Cognitive Battery on day 13 and changes in depressive symptom severity assessed with MADRS on day 13 after a series of six intravenous ketamine infusions administered within 12 days [29]. In their later study, Shiroma et al. (2020) found that improvements in speed of processing, attentional set-shifting, and spatial working memory among TRD patients were independent of improvements in depression [34]. The improvement in verbal memory was independent of a depression improvement in the study by Phillips et al. [26]. This possible pro-cognitive effect of ketamine independent of an improvement in depression has been proposed to be a clinical advantage over ECT in the acute treatment of TRD patients [34]. Additionally, it has been found that ketamine may act precisely in the cognitive domain, with a more pronounced effect on cognitive symptoms being predicted by lower default mode network deactivation and higher dorsolateral prefrontal cortex activation [38].

The possible pro-cognitive effect of esketamine illustrated by these results may be related to its impact on neuroplasticity. Based on animal studies, it has been proposed that ketamine-induced synaptic plasticity could be mediated via the mammalian target of rapamycin complex 1 (mTORC1) signaling pathway and activation of neuronal mRNA translation [39]. Additionally, a study with a mouse model of post-stroke chronic stress suggests that subanesthetic doses of S-ketamine may improve cognitive dysfunction via the inhibition of hippocampal astrocytosis [40].

Our results are subject to the inherent limitations of the small sample size, which limits the representativeness of the population. Additionally, the absence of a control intervention and a double-blind design introduces the potential for observer subjectivity bias in the assessments. To continue esketamine treatment, our patients must have shown a clinical benefit within the first month, as assessed by the psychiatrist’s clinical judgment. As a result, our patient sample has a selection bias, as all included individuals experienced an improvement in depression with esketamine. Therefore, it should be noted that the potential pro-cognitive effects of esketamine may be limited to patients with a favorable antidepressant response. The work of Sahib et al. (2020), showing increased functional activation in the supplementary motor area during a response-inhibitory task in remitters compared to non-remitters and normalizing it toward levels of activation similar to those of control subjects, illustrates that differences in processing may occur between individuals with a favorable treatment response to ketamine and those without [36]. However, except for SUSTAIN-2 [19], previous studies were short-term trials. In that sense, our study has the merit of including six months of treatment and a robust neuropsychological battery administered by an experienced neuropsychologist. It is also not possible to ensure that the improvement in cognitive performance found in our patients was not a consequence of the practice effect. However, the relatively long interval—3 months—between cognitive assessments supports the existence of an actual improvement. Nevertheless, our results should be considered hypothesis-driven and confirmed by adequately designed studies. From this perspective, further research is warranted to evaluate the long-term impact of ketamine/esketamine on cognitive function in TRD patients. Such studies should include randomized, double-blind designs with the use of active placebos to ensure robust findings.

## 4. Materials and Methods

### 4.1. Patients

We studied the severity of depression and neurocognitive performance in Brazilian, Portuguese-speaking TRD patients of both genders, aged 18 years or older, from the “Expanded Access Program” provided by the pharmaceutical company Janssen. The program consisted of the provision of treatment of an esketamine nasal spray before its marketing authorization (RESOLUTION-RDC No. 38, 12 August 2013, by the Collegiate Board of the National Health Surveillance Agency, Brazil). The program provided esketamine (SPRAVATO^®^) treatment for patients from several Brazilian sites. The included patients were from the “Expanded Access Program” provided by the pharmaceutical company Janssen, but the pharmaceutical company did not participate in the study development, or in its conceptualization, conduction, elaboration, or financial funding.

To be eligible for our study, the patient should have a diagnosis of MDD and have TRD. Treatment-resistant depression was defined as a history of an insufficient response to at least two antidepressants for more than six weeks at therapeutic doses [41]. We excluded patients with homicidal ideation/intention or suicidal ideation with some intention to act within 6 months, as judged clinically; psychotic symptoms; schizophrenia; schizoaffective disorder; bipolar disorder; intellectual disability; illicit drug use in the past 6 months; a history of ketamine/esketamine abuse; lifetime use of phencyclidine (PCP), lysergic acid diethylamide (LSD), or 3,4-methylenedioxymethamphetamine (MDMA); a history of moderate or severe alcohol use disorder; treatment with ketamine/esketamine in the past 12 months; known hypersensitivity to ketamine/esketamine; a lack of response to previous ketamine/esketamine treatment; uncontrolled systemic hypertension; clinically significant heart or lung disease; a history of hemorrhagic stroke; a history (within 6 weeks) of a cardiovascular event, including myocardial infarction; a history (within 6 months) of ischemic stroke or transient ischemic attack; uncontrolled epilepsy or seizure within 6 months; porphyria; a history or symptoms and signs suggestive of liver cirrhosis; alanine aminotransferase (ALT) or aspartate aminotransferase (AST) levels > 3.0 times the upper limit of normal; total bilirubin levels > 1.5 times the upper limit of normal; aneurysm; arteriovenous malformation; significant structural or functional abnormalities of the nose or upper airway; obstructions or lesions in the nasal mucosa (based on clinical history and nasal inspection); sinus surgery in the past 2 years; a significant deviated nasal septum that would impede effective drug absorption; and participating in another clinical research study.

Patients were recruited between February and September 2021 and treated at an outpatient unit of the Instituto de Psiquiatria, Hospital das Clínicas, Faculdade de Medicina da Universidade de São Paulo.

We invited all enrolled patients from our site’s “Expanded Access Program” after they had signed the agreement to participate in the program provided by the pharmaceutical company Janssen (RESOLUTION-RDC No. 38, 12 August 2013, by the Collegiate Board of the National Health Surveillance Agency). Those who agreed and signed the informed consent form to participate in the study were subjected to the research procedures. The Hospital das Clinicas review board approved the study.

### 4.2. Procedures

#### Treatment Protocol

The treatment protocol followed the principles used by the centers participating in the “Expanded Access Program”. Patients and their treating psychiatrist were instructed to maintain the ongoing treatment regimen, including the prescribed medications, both during and following the cessation of esketamine therapy. On the day of esketamine administration, patients were advised to have a solid food fast for at least 2 h and a liquid fast for at least 30 min before esketamine administration. The administration of esketamine occurred at the post-anesthesia recovery center within the Institute of Psychiatry. After receiving detailed instructions, the patient self-administered the esketamine nasal spray (SPRAVATO^®^) under a psychiatrist’s supervision. The nasal spray device dispensed a total of 28 mg of esketamine. For the 56 mg and 84 mg doses, the patient used 2 or 3 devices, respectively, with a 5 min interval between the use of each device. The patient remained in a hospital bed and was under continuous observation by at least one research psychiatrist throughout the entire period. We monitored the blood pressure, heart rate, and peripheral oxygen saturation during the session. The patient was required to stay in the unit for 2 h after the esketamine administration or for a more extended period, if necessary, particularly in the event of side effects or the persistence of dissociative symptoms.

The esketamine treatment protocol consisted of three distinct phases. In the initial phase, called the induction phase, patients received esketamine twice a week over a period of four weeks. The initial dose was either 56 mg or 28 mg, according to clinical judgment. Subsequently, the dose was adjusted to 56 or 84 mg according to the psychiatrist’s clinical judgment, considering both efficacy and tolerability. According to the psychiatrist’s clinical judgment, patients who exhibited a beneficial effect were moved to phase 2, the first month of the maintenance phase. In phase 2, patients received esketamine once weekly for four weeks. The dose of the induction phase was maintained in phase 2 with the flexibility for dose adjustments and increases for those still not receiving 84 mg, according to efficacy and tolerability. Following the conclusion of phase 2, patients still benefiting from esketamine were moved to phase 3. In phase 3, treatment regimens were administered either every other week or weekly, respectively, for those in remission or not, or according to clinical judgment. Patients could be maintained in phase 3 during the time indicated by their treating psychiatrists.

### 4.3. Neuropsychological Assessment

Cognitive performance was assessed at three time points. The first (T0), the baseline assessment, was performed on the first day of treatment before starting the esketamine infusion. The second and third occurred, respectively, three (T3) and six (T6) months after starting esketamine treatment. The assessment was performed by an experienced neuropsychologist (M.F.M.S.) using the neuropsychological battery described below.

#### 4.3.1. Verbal Fluency and Animal Fluency Test

The Verbal Fluency test (FAS) assesses phonemic fluency. In this test, the patient was instructed to name as many words as possible, beginning with the letters F, A, and S consecutively, during one minute for each letter. The patient could not say proper names or words or only change the endings. The score was the sum of correct words, excluding repetitions, in the three phonemic categories [42,43]. The Animal Fluency test was used to assess semantic fluency. The patient was instructed to name as many animals as possible within one minute. The score was the sum of all correct names, excluding repetitions [42,43].

#### 4.3.2. Backward Digit Span

The Backward Digit Span test assesses working memory. The examiner read aloud series of random numbers ranging from 1 to 9 with an increasing length. The patient was instructed to repeat the series in reverse sequence to that presented by the examiner. After each successful level, the list size was increased (2, 3, 4, 5, 6, 7, and 8 digits). Patients had two trials at each level, and the test ended after erring on two trials at the same level. One point was attributed for each corrected trial. The score was the sum of all correct trials, ranging from 0 to 16 [44].

#### 4.3.3. Stroop Test, Victoria Version

The Victoria version of the Stroop Test assesses executive function, cognitive flexibility, selective attention, and inhibitory control. We used it to assess inhibitory control (Stroop Color), which is considered an executive function. The test measures the ability to shift perceptual sets and simultaneous suppression of a habitual response in favor of an unusual one [43]. It consists of three parts, each using a specific card containing six rows of four items. For the first card, the Stroop Dots, the patient was instructed to name as quickly as possible the color of the 24 dots painted in green, pink, blue, and brown; it assessed the processing speed. The second card, Stroop Words, has standard and two-syllable written words; it assesses selective attention and inhibitory control. We used the Brazilian Portuguese version of the Victoria version of the Stroop Test, which included the words *cada* (each), *nunca* (never), *hoje* (today), and *tudo* (everything) printed in the colors green, pink, blue, and brown [45]. The patient was instructed to name the color in which the word was written. The third card, the Stroop Color, has color names printed in colored ink distinct from the printed name (e.g., the word “blue” printed in pink letters); the patient was instructed to name the color in which the word was printed (e.g., the word “blue” printed in pink letters the patient should answer “pink”) [46]. This is named the Stroop effect, and the Stroop Color test assesses the inhibition of cognitive interference, which is considered an executive function. For each of the three conditions, the number of errors and correct answers were recorded, as well as the time to complete each task (measured with a stopwatch). The score for each of the three test parts was the time spent performing the task, measured in seconds.

#### 4.3.4. Wisconsin Card Sorting Test

The Wisconsin Card Sorting Test (WCST) assesses the ability to develop and maintain an appropriate problem-solving strategy across changing stimulus conditions. It detects perseverative errors and requires attention, working memory, and abstract thinking. We used it to assess set-shifting, which is considered an executive function. It includes two sets of answer cards with 64 cards each and four stimulus cards. The stimulus cards include one with a red triangle, one with two green stars, one with three yellow crosses, and one with four blue circles. The stimulus cards reflect three stimulus parameters: color, shape, and number of figures. The answer cards also present pictures of different shapes, colors, and numbers. The researcher presented the four stimulus cards and instructed the patient to pick one answer card from the deck and match it with one of the four stimulus cards. The correct matching rule may be color, number of figures, or shape; the rule in use is not told to the patient. After each matching trial, the researcher informed the patient if that trial was correct or wrong. The patient should discover whether the correct matching rule was color, shape, or number of figures during the successive correct/wrong trials and match the future cards properly. After completing a sequence of ten consecutive correct responses, the rule of matching was changed (e.g., in the first sequence, the rule was the color of the figures, and the rule was changed to a number of figures in the second sequence) without warning the patient. The patient should detect the rule change and discover the new matching rule. The test was finished when the patient completed two sequences of ten correct answers for each matching rule (color, shape, and numbers) or when the cards ended. One point was assigned for each response produced by the patient. For the analysis, we included the total number of responses, the total number of errors, and the number of perseverative errors, assessing perseverative behavior and cognitive flexibility.

#### 4.3.5. Trail Making Test

The Trail Making Test (TMT) consists of two parts, A (TMT-A) and B (TMT-B), both containing 25 circles. It assesses attention (TMT-A and B), visual search (TMT-A and B), speed of processing (TMT-A and B), sequencing and shifting (TMT-B), flexibility (TMT-B), and executive functioning (TMT-B). In TMT-A, the circles contain a number (1 to 25) randomly displayed on a sheet of paper. In TMT-B, thirteen circles contain a number (1 to 13), and twelve circles contain a letter (A to L), also randomly displayed on paper. The researcher instructed the patient to draw a line connecting the numbers in ascending order in TMT-A, and in TMT-B, the line should alternate a number with a letter (i.e., 1-A; 2-B…). In both tasks, the patient was instructed to perform the tasks as quickly as possible without taking the pencil or pen off the paper. The score was the time, in seconds, taken to perform each task [43].

#### 4.3.6. Vocabulary Subtest of the WAIS-III-R (V-WAIS)

The Vocabulary Subtest (V-WAIS) of the WAIS-III-R (Wechsler Adult Intelligence Scale, Third Edition–Revised) assesses verbal and general intellectual abilities. It consists of 35 words for which patients must say their meanings. Meanings or slang not found in dictionaries are not considered correct; the researcher instructed the patient to give another meaning to those answers. A score of 2 points was given for each correct answer, a score of 1 when the patient gave some adequate information but not the main concept, and a score of 0 for a wrong answer. The total score could range from 0 to 70 points [44].

#### 4.3.7. Block Design Subtest of the WAIS-III-R

The Block Design Subtest of the WAIS-III-R assesses the intellectual ability through visual–spatial and organizational processing abilities. It comprises nine cubes with faces varying in color, some sides being red, some white, and others having a half-red and half-white pattern on the diagonal. The researcher instructed the patient to reproduce two-color models with the cubes. The degree of difficulty of the patterns was progressive, with models comprising two cubes up to the most complex ones with nine cubes. Tasks completed correctly and within the execution time limit were scored accordingly, with total scores varying from 0 to 51 [44].

#### 4.3.8. Logical Memory

Logical Memory, a subtest of the Wechsler Memory Scale (WMS), consists of two parts, LM-I and LM-II, which assess episodic and delayed episodic memory, respectively. These subtests evaluate three fundamental processes involved in memory: encoding, storage, and recall. LM-I and LM-II each consist of two stories, one featuring a woman and the other featuring a man. Each story contains 25 items (words) that the patient is required to memorize. Both stories are structured around four thematic components: character introduction, conflict, aggravating/complementary elements, and resolution [47]. The researcher read one story at a time and instructed the patient to recall it immediately after the reading. After a 30 min interval, the patient was asked to recall the stories again. The test scores were determined by the number of items recalled immediately (LM-I) and 30 min after reading (LM-II), delayed recall, with scores ranging from 0 to 50.

#### 4.3.9. Rey Auditory Verbal Learning Test

The Rey Auditory Verbal Learning test (RAVLT) assesses immediate memory span, new learning ability, susceptibility to interference, and recognition memory. Immediate memory refers to the retention of sensory information or stimuli immediately after it is perceived, and primarily involves maintaining information for immediate use. Differently from working memory, it functions as a passive storage system with minimum processing and manipulation of information.

During the test, the researcher read a list of 15 words (“list A”) to the patient and repeated it five times. After each repetition, the patient was asked to recall as many words as possible from the list A. The total number of words recalled after the first reading is called the “RAVLT immediate score”, which ranges from 0 to 15 points. The RAVLT correct total score, ranging from 0 to 75 points, is attributed to the sum of the words recalled after each of the five readings of list A. After completing the five repetitions of list A, an interference list with another 15 words was read to the patient, and he/she had to recall the words from this second list (referred to as “list B”). Subsequently, the patient was asked to recall the words from the first list A, a task denoted as “RAVLT delayed recall”. Scores for this task range from 0 to 15 points. After 30 min, the patient was asked again to recall the words from the first list (list A), which is referred to as “RAVLT 30 min recall”, with scores ranging from 0 to 15 points. Following this, a sheet containing 50 words was presented to the patient; 30 words were those from lists A and B, and the 20 additional words were phonetically and semantically similar to those from lists A and B; the patient was asked to recognize which of those words were from list A. This task, named “RAVLT recognition” ranges from 0–15 points [43].

#### 4.3.10. Digit Symbol-Coding

The Digit Symbol-Coding subtest of the WAIS-III-R assesses mental processing speed, attention, clerical efficiency, and visual–motor coordination [44]. The test comprises a model featuring a numerical sequence from 1 to 9, with a corresponding symbol beneath each number. Within the task component of the test, 140 numbers ranging from 1 to 9 are arranged in a random sequence. Beneath each number, a space is designated for reproducing the corresponding symbol. The researcher instructed the patient to replicate the symbol associated with each number as quickly as possible. The task concluded after 120 s. One point was awarded for each accurately reproduced symbol, resulting in a score ranging from 0 to 133 points.

### 4.4. Depression Assessment

A psychiatrist diagnosed TRD in an open clinical interview. To be considered resistant to treatment, the patient should have a diagnosis of major depressive disorder (MDD) according to the DSM-5 criteria [48] and presented an insufficient response to at least two different classes of antidepressants after more than six weeks of treatment at therapeutic doses [41]. The severity of depression was assessed with the Montgomery–Åsberg Depression Rating Scale (MADRS). The MADRS is a clinician-rated scale comprised of 10 items; each item’s severity ranges from 0 to 60 [49]. MADRS total scores range from 0 to 60, with higher scores reflecting greater depression severity. A psychiatrist applied the MADRS before each esketamine administration.

### 4.5. Analysis

The main purpose of this study was to investigate the effect of six months of esketamine nasal spray treatment as adjunct treatment on the cognitive performance of TRD patients. Patients were subjected to neuropsychological assessments at T0 (before starting treatment), T3 (three months of treatment), and T6 (six months of treatment). To test the changes in scores by comparing the three time points, considering the small sample size and that we are dealing with repeated measures, we opted to utilize a method originally developed for comparing measurements and assessing bias, precision, and reliability. This procedure is typically used when one intends to replace one measurement technique with another. The situation here is analogous, except that the measurement technique is the same but applied on two distinct occasions, and so the assessment of the discrepancy between the initial and final values, which is undesirable in the case of measurement techniques, here represents an evaluation of the extent of the change in the score value on average, corresponding to the accuracy test that assesses the bias between the two time points [50] using the R package eirasagree version 5, available at Harvard Dataverse [51].

Two strategies were employed to test the association between the MADRS total scores and the score of each of the neuropsychological tests. The first was a correlation measure with correction for repeated measures [52] implemented in R using the function rmcorr from the rmcorr package [53]. The second strategy involves a regression analysis obtained from a general linear mixed model (GLMM), where the patient is used as their own control (random effect) due to the repeated measures. This regression was implemented with the function lmer from the R package lmer Test [54].

All three measurement points can be considered simultaneously using correlation and regression analyses. In addition to investigating changes from T0 to T6, we conducted separate analyses specifically addressing changes from T0 to T3 and from T3 to T6. This approach allowed us to identify changes that occurred specifically in the first three months, changes that occurred especially in the last three months, and changes that occurred during both periods. Thus, to pinpoint when the change occurred, we examined the regression analyses for the intervals from T0 to T6, T0 to T3, and T3 to T6. For the interference analysis, we used the Tukey methods to compare the three estimates (T0–T3; T0–T6 and T3–T6) after we used Dunnett methods for two estimates (T0–T3 and T0–T6). The confidence level was 0.95, and Bonferroni methods adjusted the *p*-value < 0.05. The analysis was adjusted by gender, age, and educational level as independent variables.

## 5. Conclusions

In conclusion, the results of our report of eight TRD patients suggest that patients that have experienced an improvement in depression with intranasal esketamine used as an adjunct antidepressant treatment for six months may have improvements in cognitive functions of attention, memory, and the executive functions of working memory, set-shifting, and inhibitory control. Also, most functions may already achieve improvements at three months of treatment. Still, some functions may continuously improve throughout the six months, while others may require more than three months to improve significantly, and some functions that improved at three months may not sustain the improvement at six months of esketamine treatment. Additionally, improvements in cognitive functions are mostly correlated with the amelioration of depression. Our results did not suggest any possible harm from intranasal esketamine treatment. The only decrease in performance found in processing speed from T3 to T6 was counterbalanced by a previous significant improvement from T0 to T3. Our results highlight the relevance of studies adequately designed to investigate the potential neurocognitive effects of esketamine used as an adjunct antidepressant for TRD.

## Figures and Tables

**Table 1:** Sociodemographic characteristics of TRD patients treated with the esketamine nasal spray as adjunct treatment.

Patient	Sex	Age (Years)	Education Level	Marital Status	Employment Status
01	male	55	15	single	working
02	female	39	18	divorced	working
03	male	39	13	single	working
04	male	44	05	maried	not working
05	male	76	11	maried	not working
06 (*)	female	40	15	single	not working
07	female	56	17	maried	working
08	female	42	17	single	working
09 (*)	male	55	13	single	working
10	female	46	17	maried	not working
11 (*)	male	45	15	maried	working
12 (*)	male	29	18	single	working

TRD = treatment-resistant depression. (*) Patients not subjected to a second neuropsychological assessment. Educ. Level = education level (years of study).

**Table 2:** Severity of depression and cognitive performance at baseline and after three and six months of adjunct esketamine treatment.

	T0 n	Mean (sd)		Median (min, max)	T3 n	Mean (sd)	Median (min, max)	T6 n	Mean (sd)	Median (min, max)
**MADRS TOTAL**	10	28.4	(9.6)	31 (12, 42)	9	12.3 (9.2)	11 (1, 25)	7	7.7 (3.5)	8 (4, 14)
**Stroop Dots**	12	16.8	(8.6)	13 (11, 40)	8	16.3 (6.1)	18 (9, 28)	8	19.5 (5.5)	18 (12, 30)
**Stroop Words**	12	19.0	(7.0)	18 (12, 34)	8	18.0 (5.6)	18 (10, 25)	8	19.3 (6.8)	18 (12, 30)
**Stroop Color**	12	29.4	(10.5)	27 (15, 45)	8	26.1 (6.7)	24 (19, 37)	8	25.8 (7.9)	25 (18, 37)
**TMT-A**	12	39.5	(16.6)	35 (21, 75)	8	32.5 (11.4)	34 (18, 50)	8	29.4 (7.9)	31 (19, 40)
**TMT-B**	12	72.8	(33.6)	74 (2, 125)	8	54.4 (39.5)	59 (1, 109)	8	67.1 (36.0)	75 (1, 100)
**Digit Symbol-Coding**	12	61.7	(17.4)	67 (29, 85)	8	72.5 (12.3)	74 (50, 88)	8	64.1 (19.0)	66 (32, 93)
**RAVLT, Correct**	12	52.9	(15.5)	55 (27, 85)	8	64.8 (13.8)	64 (44, 85)	8	68.8 (10.2)	70 (56, 85)
**RAVLT, Immediate**	12	4.8	(2.7)	5 (1, 12)	8	5.5 (1.6)	6 (3, 8)	8	6.8 (1.8)	7 (4, 10)
**RAVLT, 30 Min. Recall**	12	7.9	(4.0)	8 (0, 15)	7	10.0 (3.8)	10 (5, 14)	7	9.0 (4.0)	10 (3, 14)
**RAVLT, Delayed Recall**	12	9.2	(2.9)	9 (5, 15)	8	10.4 (3.0)	10 (6, 15)	8	9.6 (3.8)	11 (3, 14)
**RAVLT, Recognition**	12	13.1	(2.1)	14 (9, 15)	7	13.0 (1.8)	14 (10, 15)	7	13.3 (1.5)	13 (11, 15)
**Logical Memory I**	12	40.5	(22.4)	41 (0, 90)	8	53.0 (24.5)	48 (16, 96)	8	54.0 (25.1)	53 (22, 100)
**Logical Memory II**	12	31.7	(24.3)	25 (0, 96)	8	49.8 (26.3)	46 (6, 100)	8	45.3 (29.2)	45 (8, 100)
**Category Animals Fluency**	7	17.0	(4.2)	18 (10, 22)	8	16.5 (5.3)	19 (8, 23)	8	17.6 (2.7)	18 (14, 21)
**FAS Test**	12	37.9	(10.0)	38 (24, 57)	8	42.5 (7.9)	43 (33, 54)	8	40.9 (8.5)	40 (31, 56)
**Digit Span Backward**	12	5.8	(1.7)	7 (3, 8)	8	6.0 (1.9)	6 (3, 9)	8	7.6 (2.2)	7 (5, 11)
**WCST, Total Cards**	12	72.3	(16.6)	71 (48, 100)	8	73.6 (16.9)	80 (43, 89)	8	79.4 (15.7)	83 (52, 98)
**WCST, Total Correct**	12	103.9	(26.4)	112 (60, 128)	8	94.8 (23.4)	84 (71, 128)	8	90.4 (25.7)	82 (61, 128)
**WCST, Total Errors**	12	32.4	(23.4)	33 (0, 66)	8	27.8 (25.2)	17 (8, 73)	8	22.1 (21.8)	15 (1, 61)
**WCST, Perseverative Errors**	12	12.8	(13.4)	12 (0, 47)	8	9.3 (13.1)	5 (0, 39)	8	6.6 (10.7)	2 (0, 30)
**Block, WAIS-III-R**	12	24.0	(13.1)	22 (6, 46)	8	24.1 (14.7)	24 (4, 46)	8	22.0 (16.2)	19 (2, 51)
**Vocabulary, WAIS-III-** **R**	12	47.9	(15.4)	52 (2, 61)	8	51.8 (10.0)	55 (28, 61)	8	54.1 (10.4)	59 (35, 63)

T0 = baseline assessment; T3 = assessment at three months of adjunct esketamine treatment; T6 = assessment at six months of adjunct esketamine treatment. FAS = Verbal Fluency Test; MADRS = Montgomery–Åsberg Depression Rating Scale; RAVLT = Rey Auditory Verbal Learning Test; TMT-A = Trail Making Test-A; TMT-B = Trail Making Test-B; WAIS-III-R = Wechsler Adult Intelligence Scale, Third Edition–Revised; WCST = Wisconsin Card Sorting Test.

**Table 3:** Comparisons of cognitive performance and the severity of depression between time points. T0–T6 interval.

T0 × T6	Test	n	bias	CI95	Decision
	MADRS TOTAL	7	−20.99	[−25.30, −17.36]	improvement
	Stroop Dots	8	1.57	[−8.60, 6.20]	
	Stroop Words	8	−0.24	[−3.16, 6.84	
	Stroop Color	8	−6.46	[−9.68, −4.11	improvement
	TMT-A	8	−14.27	[−17.44, −10.40]	improvement
	TMT-B	8	−4.50	[−34.31, 21.52]	
	Digit Symbol-Coding	8	2.65	[−4.17, 7.67]	
	RAVLT, Correct	8	18.83	[11.29, 29.61]	improvement
	RAVLT, Immediate	8	1.87	[0.56, 4.11]	improvement
	RAVLT, 30 Min. Recall	7	2.61	[−1.09, 4.68]	
	RAVLT, Delayed Recall	8	0.78	[−1.65, 3.10]	
	RAVLT, Recognition	7	1.27	[−1.00, 2.67]	
	Logical Memory I	8	11.67	[−6.46, 21.72]	
	Logical Memory II	8	11.78	[4.88, 19.31]	improvement
	Category Animals Fluency	5	0.01	[−4.15, 1.80]	
	FAS Test	8	2.32	[−4.27, 6.35]	
	Digit Span Backward	8	2.13	[0.88, 3.45]	improvement
	WCST, Total Cards	8	11.07	[−4.95, 19.56]	
	WCST, Total Correct	8	−19.11	[−32.00, −4.89]	improvement
	WCST, Total Errors	8	−16.14	[−28.68, 5.41]	
	WCST, Perseverative Errors	8	−8.31	[−13.35, 4.39]	
	Block, WAIS-III-R	8	−0.92	[−8.20, 4.97]	
	Vocabulary, WAIS-III-R	8	8.80	[−5.29, 13.63]	

The statistical analysis of changes in the mean score (bias) between time points (T0, T3, and T6) was conducted using bootstrap resampling (5000 iterations). Statistical significance was determined when the null difference was not included within the 95% confidence interval (CI95). T0 = baseline assessment; T3 = assessment at three months of adjunct esketamine treatment; T6 = assessment at six months of adjunct esketamine treatment. FAS = Verbal Fluency Test; MADRS = Montgomery–Åsberg Depression Rating Scale; RAVLT = Rey Auditory Verbal Learning Test; TMT-A = Trail Making Test-A; TMT-B = Trail Making Test-B; WAIS-III-R = Wechsler Adult Intelligence Scale, Third Edition–Revised; WCST = Wisconsin Card Sorting Test.

**Table 4:** Comparisons of cognitive performance and the severity of depression between time points. T0–T3 interval.

T0 × T3	Test	n	bias	CI95	Decision
	MADRS TOTAL	9	−18.60	[−26.52, −11.32]	improvement
	Stroop Dots	8	−1.59	[−3.61, 1.98]	
	Stroop Words	8	−1.58	[−3.40, 2.02]	
	Stroop Color	8	−4.98	[−8.25, −1.40]	improvement
	TMT-A	8	−7.50	[−14.76, −1.00]	improvement
	TMT-B	8	−25.41	[−58.25, 3.13]	
	Digit Symbol-Coding	8	7.71	[4.42, 11.07]	improvement
	RAVLT, Correct	8	17.54	[7.57, 29.72]	improvement
	RAVLT, Immediate	8	1.10	[−0.06, 2.90]	
	RAVLT, 30 Min. Recall	7	3.30	[1.39, 5.02]	improvement
	RAVLT, Delayed Recall	8	1.25	[−0.50, 3.23]	
	RAVLT, Recognition	7	0.97	[−2.00, 2.37]	
	Logical Memory I	8	13.77	[4.68, 25.30]	improvement
	Logical Memory II	8	15.47	[8.70, 23.04]	improvement
	Category Animals Fluency	4	0.71	[−2.60, 4.01]	
	FAS Test	8	3.56	[−1.33, 7.52]	
	Digit Span Backward	8	0.07	[−0.91, 1.13]	
	WCST, Total Cards	8	2.33	[−13.84, 12.53]	
	WCST, Total Correct	8	−8.47	[−23.12, 5.56]	
	WCST, Total Errors	8	−5.92	[−21.56, 20.03]	
	WCST, Perseverative Errors	8	−4.48	[−10.54, −0.02]	improvement
	Block, WAIS-III-R	8	1.42	[−3.09, 9.22]	
	Vocabulary, WAIS-III-R	8	6.20	[1.98, 11.31]	improvement

The statistical analysis of changes in the mean score (bias) between time points (T0, T3, and T6) was conducted using bootstrap resampling (5000 iterations). Statistical significance was determined when the null difference was not included within the 95% confidence interval (CI95). T0 = baseline assessment; T3 = assessment at three months of adjunct esketamine treatment; T6 = assessment at six months of adjunct esketamine treatment. FAS = Verbal Fluency Test; MADRS = Montgomery–Åsberg Depression Rating Scale; RAVLT = Rey Auditory Verbal Learning Test; TMT-A = Trail Making Test-A; TMT-B = Trail Making Test-B; WAIS-III-R = Wechsler Adult Intelligence Scale, Third Edition–Revised; WCST = Wisconsin Card Sorting Test.

**Table 5:** Comparisons of cognitive performance and the severity of depression between time points. T3–T6 interval.

T3 × T6	Test	n	bias	CI95	Decision
	MADRS TOTAL	7	−4.71	[−8.49, −1.98]	improvement
	Stroop Dots	7	3.30	[−0.81, 8.65]	
	Stroop Words	7	1.56	[−0.59, 3.61]	
	Stroop Color	7	0.01	[−3.31, 5.42]	
	TMT-A	7	−5.70	[−9.49, −2.45]	improvement
	TMT-B	7	7.30	[−7.72, 35.68]	
	Digit Symbol-Coding	7	−6.44	[−10.79, −2.34]	worsening
	RAVLT, Correct	7	4.09	[−2.60, 10.32]	
	RAVLT, Immediate	7	1.50	[0.33, 2.57]	improvement
	RAVLT, 30 Min. Recall	6	−0.44	[−7.00, 1.44]	
	RAVLT, Delayed Recall	7	−0.26	[−1.58, 1.36]	
	RAVLT, Recognition	6	−0.23	[−2.00, 0.67]	
	Logical Memory I	7	4.61	[−4.42, 11.92]	
	Logical Memory II	7	−1.16	[−8.94, 3.17]	
	Category Animals Fluency	7	1.49	[−1.53, 3.16]	
	FAS Test	7	−1.22	[−9.77, 1.83]	
	Digit Span Backward	7	1.84	[0.00, 4.09]	improvement
	WCST, Total Cards	7	8.49	[2.69, 15.74]	improvement
	WCST, Total Correct	7	−7.00	[−22.64, 3.09]	
	WCST, Total Errors	7	−8.43	[−20.48, −2.98]	improvement
	WCST, Perseverative Errors	7	−2.71	[−10.10, 8.81]	
	Block, WAIS-III-R	7	−0.45	[−8.51, 4.52]	
	Vocabulary, WAIS-III-R	7	5.58	[2.19, 11.74]	improvement

The statistical analysis of changes in the mean score (bias) between time points (T0, T3, and T6) was conducted using bootstrap resampling (5000 iterations). Statistical significance was determined when the null difference was not included within the 95% confidence interval (CI95). T0 = baseline assessment; T3 = assessment at three months of adjunct esketamine treatment; T6 = assessment at six months of adjunct esketamine treatment. FAS = Verbal Fluency Test; MADRS = Montgomery–Åsberg Depression Rating Scale; RAVLT = Rey Auditory Verbal Learning Test; TMT-A = Trail Making Test-A; TMT-B = Trail Making Test-B; WAIS-III-R = Wechsler Adult Intelligence Scale, Third Edition–Revised; WCST = Wisconsin Card Sorting Test.

**Table 6:** Associations between total MADRS scores and neuropsychological tests, global.

	R.M. Correlation				GLMM			
Global	*n*	r	CI95	*p*	Slope	CI95	t (df)	*p*
Stroop Dots	15	0.079	[−0.434, 0.553]	0.771	0.020	[−0.206, 0.247]	0.19 (18.9)	0.854
Stroop Words	15	0.432	[−0.082, 0.764]	0.095	0.086	[−0.027, 0.198]	1.62 (15.3)	0.125
Stroop Color	15	0.694	[0.303, 0.885]	0.003 *	0.245	[0.087, 0.402]	3.31 (15.0)	0.005 *
TMT-A	15	0.771	[0.446, 0.917]	4.7 × 10^−4^ *	0.519	[0.282, 0.756]	4.68 (14.4)	3.3× 10^−4^ *
TMT-B	15	0.140	[−0.382, 0.594]	0.605	0.011	[−1.191, 1.213]	0.02 (17.0)	0.985
Digit Symbol-Coding	15	−0.424	[−0.760, 0.090]	0.101	−0.212	[−0.459, 0.035]	−1.83 (14.5)	0.088
RAVLT, Correct	15	−0.526	[−0.810, −0.041]	0.036 *	−0.589	[−1.063, −0.116]	−2.60 (19.9)	0.017 *
RAVLT, Immediate	15	−0.301	[−0.693, 0.229]	0.258	−0.053	[−0.129, 0.023]	−1.45 (19.7)	0.162
RAVLT, 30 Min. Recall	13	−0.599	[−0.857, −0.100]	0.024 *	−0.108	[−0.201, −0.014]	−2.47 (14.3)	0.027 *
RAVLT, Delayed Recall	15	−0.192	[−0.628, 0.336]	0.476	−0.034	[−0.111, 0.043]	−0.93 (16.9)	0.363
RAVLT, Recognition	13	−0.435	[−0.784, 0.125]	0.120	−0.039	[−0.101, 0.022]	−1.34 (18.3)	0.196
Logical Memory I	15	−0.788	[−0.923, −0.480]	2.9 × 10^−4^ *	−0.717	[−1.039, −0.395]	−4.75 (14.8)	2.7× 10^−4^ *
Logical Memory II	15	−0.784	[−0.922, −0.471]	3.3 × 10^−4^ *	−0.610	[−0.883, −0.337]	−4.77 (14.5)	2.7× 10^−4^ *
Category Animals Fluency	11	−0.024	[−0.590, 0.557]	0.941	0.016	[−0.151, 0.183]	0.21 (14.2)	0.839
FAS Test	15	−0.314	[−0.700, 0.216]	0.237	−0.085	[−0.256, 0.085]	−1.06 (15.0)	0.304
Digit Span Backward	15	−0.574	[−0.833, −0.110]	0.020 *	−0.068	[−0.121, −0.015]	−2.70 (17.5)	0.015 *
WCST, Total Cards	15	−0.092	[−0.562, 0.423]	0.735	−0.025	[−0.487, 0.437]	−0.11 (17.5)	0.911
WCST, Total Correct	15	0.221	[−0.308, 0.646]	0.410	0.237	[−0.399, 0.874]	0.79 (16.2)	0.441
WCST, Total Errors	15	0.120	[−0.399, 0.581]	0.657	0.076	[−0.572, 0.725]	0.25 (17.4)	0.807
WCST, Perseverative Errors	15	0.315	[−0.214, 0.701]	0.234	0.150	[−0.169, 0.469]	0.99 (16.4)	0.334
Block, WAIS-III-R	15	−0.033	[−0.520, 0.471]	0.904	−0.009	[−0.196, 0.178]	−0.10 (14.9)	0.920
Vocabulary, WAIS-III-R	15	−0.657	[−0.869, −0.239]	0.006 *	−0.379	[−0.624, −0.134]	−3.27 (15.9)	0.005 *

R.M. correlations = the analysis was estimated by a repeated measures correlation (R function), GLMM = regression by a general linear mixed model (GLMM, R function). * Indicates *p* values smaller than the significance level of 0.05. T0 = baseline assessment; T3 = assessment at three months of adjunct esketamine treatment; T6 = assessment at six months of adjunct esketamine treatment. FAS = Verbal Fluency test; MADRS = Montgomery–Åsberg Depression Rating Scale; RAVLT = Rey Auditory Verbal Learning Test; TMT-A = Trail Making Test-A; TMT-B = Trail Making Test-B; WAIS-III-R = Wechsler Adult Intelligence Scale, Third Edition–Revised; WCST = Wisconsin Card Sorting Test.

**Table 7:** Associations between total MADRS scores and neuropsychological tests, T0–T6 interval.

	R.M. Correlation				GLMM			
T0 × T6	*n*	r	CI95	*p*	Slope	CI95	t (df)	*p*
Stroop Dots	7	−0.074	[−0.740, 0.666]	>0.995	−0.020	[−0.340, 0.300]	−0.13 (11.0)	>0.995
Stroop Words	7	0.297	[−0.515, 0.828]	>0.995	0.049	[−0.138, 0.237]	0.61 (8.1)	>0.995
Stroop Color	7	0.823	[0.283, 0.967]	0.036 *	0.250	[0.061, 0.439]	3.19 (6.4)	0.052
TMT-A	7	0.874	[0.440, 0.977]	0.014 *	0.577	[0.278, 0.877]	4.63 (6.5)	0.009 *
TMT-B	7	0.503	[−0.312, 0.892]	0.612	0.361	[−1.123, 1.844]	0.59 (6.3)	>0.995
Digit Symbol-Coding	7	−0.434	[−0.872, 0.390]	0.849	−0.096	[−0.288, 0.096]	−1.21 (6.2)	0.809
RAVLT, Correct	7	−0.715	[−0.944, −0.020]	0.139	−0.677	[−1.273, −0.080]	−2.50 (10.8)	0.089
RAVLT, Immediate	7	−0.519	[−0.896, 0.292]	0.561	−0.081	[−0.183, 0.021]	−1.76 (10.8)	0.321
RAVLT, 30 Min. Recall	6	−0.825	[−0.973, −0.190]	0.067	−0.128	[−0.228, −0.028]	−3.12 (6.1)	0.061
RAVLT, Delayed Recall	7	−0.285	[−0.824, 0.525]	>0.995	−0.042	[−0.154, 0.070]	−0.84 (9.7)	>0.995
RAVLT, Recognition	6	−0.712	[−0.954, 0.088]	0.218	−0.049	[−0.121, 0.023]	−1.57 (7.9)	0.470
Logical Memory I	7	−0.970	[−0.995, −0.838]	2.0 × 10^−4^ *	−0.841	[−1.050, −0.631]	−9.75 (6.2)	1.7 × 10^−4^ *
Logical Memory II	7	−0.904	[−0.983, −0.549]	0.006 *	−0.604	[−0.884, −0.325]	−5.23 (6.3)	0.005 *
Category Animals Fluency	4	0.668	[−0.522, 0.975]	0.655	0.066	[−0.083, 0.215]	1.29 (3.6)	0.822
FAS Test	7	−0.078	[−0.742, 0.663]	>0.995	−0.013	[−0.271, 0.245]	−0.12 (7.7)	>0.995
Digit Span Backward	7	−0.818	[−0.966, −0.266]	0.040 *	−0.085	[−0.145, −0.026]	−3.35 (7.7)	0.032 *
WCST, Total Cards	7	−0.339	[−0.842, 0.480]	>0.995	−0.083	[−0.735, 0.569]	−0.29 (9.4)	>0.995
WCST, Total Correct	7	0.362	[−0.460, 0.850]	>0.995	0.279	[−0.647, 1.204]	0.69 (8.5)	>0.995
WCST, Total Errors	7	0.293	[−0.518, 0.827]	>0.995	0.084	[−0.847, 1.014]	0.20 (9.8)	>0.995
WCST, Perseverative Errors	7	0.367	[−0.455, 0.852]	>0.995	0.115	[−0.387, 0.618]	0.51 (9.6)	>0.995
Block, WAIS-III-R	7	0.117	[−0.640, 0.759]	>0.995	0.024	[−0.146, 0.195]	0.34 (6.4)	>0.995
Vocabulary, WAIS-III-R	7	−0.840	[−0.970, −0.331]	0.027 *	−0.515	[−0.835, −0.196]	−3.77 (7.4)	0.019 *

R.M. correlations = the analysis was estimated by a repeated measures correlation (R function), GLMM = regression by a general linear mixed model (GLMM, R function). * Indicates *p* values smaller than the significance level of 0.05. T0 = baseline assessment; T3 = assessment at three months of adjunct esketamine treatment; T6 = assessment at six months of adjunct esketamine treatment. FAS = Verbal Fluency test; MADRS = Montgomery–Åsberg Depression Rating Scale; RAVLT = Rey Auditory Verbal Learning Test; TMT-A = Trail Making Test-A; TMT-B = Trail Making Test-B; WAIS-III-R = Wechsler Adult Intelligence Scale, Third Edition–Revised; WCST = Wisconsin Card Sorting Test.

**Table 8:** Associations between total MADRS scores and neuropsychological tests, T0–T3 interval.

	R.M. Correlation				GLMM			
T0 × T3	*n*	r	CI95	*p*	Slope	CI95	t (df)	*p*
Stroop Dots	8	0.499	[−0.247, 0.874]	0.514	0.135	[−0.068, 0.338]	1.52 (8.3)	0.498
Stroop Words	8	0.655	[−0.016, 0.919]	0.166	0.128	[−0.007, 0.262]	2.20 (7.8)	0.181
Stroop Color	8	0.624	[−0.069, 0.911]	0.218	0.209	[−0.078, 0.496]	1.66 (8.5)	0.398
TMT-A	8	0.838	[0.392, 0.965]	0.014 *	0.499	[0.218, 0.780]	4.17 (7.3)	0.012 *
TMT-B	8	0.116	[−0.594, 0.724]	>0.995	−0.235	[−1.970, 1.499]	−0.29 (12.4)	>0.995
Digit Symbol-Coding	8	−0.808	[−0.958, −0.310]	0.025 *	−0.425	[−0.698, −0.153]	−3.67 (7.3)	0.023 *
RAVLT, Correct	8	−0.452	[−0.858, 0.303]	0.665	−0.502	[−1.176, 0.172]	−1.61 (13.0)	0.395
RAVLT, Immediate	8	−0.069	[−0.701, 0.624]	>0.995	−0.023	[−0.126, 0.081]	−0.48 (12.4)	>0.995
RAVLT, 30 Min. Recall	7	−0.639	[−0.926, 0.120]	0.265	−0.123	[−0.268, 0.021]	−1.96 (8.2)	0.254
RAVLT, Delayed Recall	8	−0.153	[−0.742, 0.569]	>0.995	−0.039	[−0.147, 0.070]	−0.79 (10.0)	>0.995
RAVLT, Recognition	7	−0.388	[−0.858, 0.436]	>0.995	−0.048	[−0.138, 0.042]	−1.13 (15.0)	0.827
Logical Memory I	8	−0.729	[−0.939, −0.127]	0.077	−0.671	[−1.225, −0.117]	−2.80 (8.0)	0.070
Logical Memory II	8	−0.783	[−0.952, −0.249]	0.037 *	−0.705	[−1.186, −0.225]	−3.41 (7.6)	0.029*
Category Animals Fluency	4	−0.412	[−0.949, 0.739]	>0.995	−0.015	[−0.262, 0.233]	−0.17 (3.6)	>0.995
FAS Test	8	−0.685	[−0.927, −0.038]	0.125	−0.195	[−0.403, 0.013]	−2.19 (7.4)	0.188
Digit Span Backward	8	−0.215	[−0.769, 0.524]	>0.995	−0.019	[−0.069, 0.032]	−0.84 (8.4)	>0.995
WCST, Total Cards	8	0.275	[−0.476, 0.794]	>0.995	0.286	[−0.344, 0.916]	1.00 (10.9)	>0.995
WCST, Total Correct	8	−0.023	[−0.677, 0.651]	>0.995	−0.067	[−1.003, 0.870]	−0.16 (9.7)	>0.995
WCST, Total Errors	8	−0.164	[−0.747, 0.561]	>0.995	−0.293	[−1.223, 0.637]	−0.69 (11.0)	>0.995
WCST, Perseverative Errors	8	0.319	[−0.438, 0.811]	>0.995	0.086	[−0.198, 0.370]	0.70 (7.7)	>0.995
Block, WAIS-III-R	8	0.005	[−0.662, 0.667]	>0.995	−0.005	[−0.305, 0.296]	−0.03 (8.1)	>0.995
Vocabulary, WAIS-III-R	8	−0.517	[−0.879, 0.224]	0.463	−0.274	[−0.641, 0.093]	−1.70 (8.7)	0.376

R.M. correlations = the analysis was estimated by a repeated measures correlation (R function), GLMM = regression by a general linear mixed model (GLMM, R function). * Indicates *p* values smaller than the significance level of 0.05. T0 = baseline assessment; T3 = assessment at three months of adjunct esketamine treatment; T6 = assessment at six months of adjunct esketamine treatment. FAS = Verbal Fluency test; MADRS = Montgomery–Åsberg Depression Rating Scale; RAVLT = Rey Auditory Verbal Learning Test; TMT-A = Trail Making Test-A; TMT-B = Trail Making Test-B; WAIS-III-R = Wechsler Adult Intelligence Scale, Third Edition–Revised; WCST = Wisconsin Card Sorting Test.

**Table 9:** Associations between total MADRS scores and neuropsychological tests, T3–T6 interval.

	R.M. Correlation				GLMM			
T3 × T6	*n*	r	CI95	*p*	Slope	CI95	t (df)	*p*
Stroop Dots	7	−0.047	[−0.728, 0.680]	>0.995	−0.152	[−0.640, 0.336]	−0.67 (13.0)	>0.995
Stroop Words	7	0.236	[−0.562, 0.807]	>0.995	0.029	[−0.223, 0.281]	0.27 (7.1)	>0.995
Stroop Color	7	0.609	[−0.168, 0.919]	0.328	0.182	[−0.120, 0.485]	1.44 (6.7)	0.587
TMT-A	7	0.204	[−0.585, 0.794]	>0.995	0.035	[−0.603, 0.674]	0.12 (9.8)	>0.995
TMT-B	7	−0.632	[−0.925, 0.132]	0.279	−2.045	[−4.260, 0.170]	−2.07 (9.5)	0.200
Digit Symbol-Coding	7	0.222	[−0.572, 0.801]	>0.995	0.187	[−0.632, 1.005]	0.52 (8.2)	>0.995
RAVLT, Correct	7	0.149	[−0.621, 0.772]	>0.995	−0.078	[−0.894, 0.738]	−0.22 (9.5)	>0.995
RAVLT, Immediate	7	−0.084	[−0.745, 0.660]	>0.995	−0.027	[−0.163, 0.109]	−0.44 (11.3)	>0.995
RAVLT, 30 Min. Recall	6	0.219	[−0.639, 0.835]	>0.995	0.037	[−0.186, 0.261]	0.39 (7.9)	>0.995
RAVLT, Delayed Recall	7	0.000	[−0.705, 0.705]	>0.995	−0.002	[−0.139, 0.136]	−0.03 (7.5)	>0.995
RAVLT, Recognition	6	0.149	[−0.680, 0.811]	>0.995	0.005	[−0.095, 0.104]	0.11 (7.0)	>0.995
Logical Memory I	7	−0.225	[−0.803, 0.570]	>0.995	−0.201	[−1.098, 0.696]	−0.53 (7.2)	>0.995
Logical Memory II	7	−0.194	[−0.790, 0.592]	>0.995	−0.109	[−0.668, 0.450]	−0.47 (6.3)	>0.995
Category Animals Fluency	7	−0.070	[−0.738, 0.668]	>0.995	0.073	[−0.231, 0.377]	0.53 (10.7)	>0.995
FAS Test	7	0.343	[−0.477, 0.844]	>0.995	0.171	[−0.144, 0.486]	1.28 (7.0)	0.723
Digit Span Backward	7	−0.870	[−0.976, −0.426]	0.015 *	−0.196	[−0.315, −0.078]	−3.87 (7.5)	0.016 *
WCST, Total Cards	7	−0.518	[−0.896, 0.294]	0.565	−0.379	[−1.139, 0.382]	−1.17 (7.2)	0.837
WCST, Total Correct	7	0.555	[−0.246, 0.905]	0.461	0.612	[−0.427, 1.651]	1.39 (7.1)	0.621
WCST, Total Errors	7	0.667	[−0.071, 0.933]	0.213	0.615	[−0.131, 1.361]	1.98 (6.5)	0.275
WCST, Perseverative Errors	7	0.294	[−0.518, 0.827]	>0.995	0.062	[−0.749, 0.874]	0.17 (9.8)	>0.995
Block, WAIS-III-R	7	−0.292	[−0.827, 0.520]	>0.995	−0.169	[−0.757, 0.419]	−0.68 (7.2)	>0.995
Vocabulary, WAIS-III-R	7	−0.217	[−0.799, 0.576]	>0.995	−0.096	[−0.594, 0.401]	−0.44 (8.2)	>0.995

R.M. correlations = the analysis was estimated by a repeated measures correlation (R function), GLMM = regression by a general linear mixed model (GLMM, R function). * Indicates *p* values smaller than the significance level of 0.05. T0 = baseline assessment; T3 = assessment at three months of adjunct esketamine treatment; T6 = assessment at six months of adjunct esketamine treatment. FAS = Verbal Fluency test; MADRS = Montgomery–Åsberg Depression Rating Scale; RAVLT = Rey Auditory Verbal Learning Test; TMT-A = Trail Making Test-A; TMT-B = Trail Making Test-B; WAIS-III-R = Wechsler Adult Intelligence Scale, Third Edition–Revised; WCST = Wisconsin Card Sorting Test.

## Data Availability

The data can be obtained by contacting Prof. Fraguas directly at renerio.fraguas@fm.usp.br.
